# Aerobic Capacity, Activity Levels and Daily Energy Expenditure in Male and Female Adolescents of the Kenyan Nandi Sub-Group

**DOI:** 10.1371/journal.pone.0066552

**Published:** 2013-06-21

**Authors:** Alexander R. Gibson, Robert Ojiambo, Kenn Konstabel, Daniel E. Lieberman, John J. Reilly, John R. Speakman, Yannis P. Pitsiladis

**Affiliations:** 1 College of Medicine, Veterinary and Life Sciences, Institute of Cardiovascular and Medical Sciences, University of Glasgow, West Medical Building, Glasgow, Scotland; 2 Department of Medical Physiology, Moi University, Eldoret, Kenya; 3 Institute of Psychology, University of Tartu, Tartu, Estonia; 4 Physical Activity for Health Group, School of Psychological Science, University of Tartu, Tartu, Estonia; 5 Department of Human Evolutionary Biology, Harvard University, Cambridge, Massachusetts, United States of America; 6 Institute of Biological and Environmental Sciences, University of Aberdeen, Aberdeen, Scotland; 7 Institute of Genetics and Developmental Biology, Key State Laboratory of Molecular Developmental Biology, Chinese Academy of Sciences, Beijing, People’s Republic of China; NIDDK/NIH, United States of America

## Abstract

The relative importance of genetic and socio-cultural influences contributing to the success of east Africans in endurance athletics remains unknown in part because the pre-training phenotype of this population remains incompletely assessed. Here cardiopulmonary fitness, physical activity levels, distance travelled to school and daily energy expenditure in 15 habitually active male (13.9±1.6 years) and 15 habitually active female (13.9±1.2) adolescents from a rural Nandi primary school are assessed. Aerobic capacity (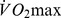) was evaluated during two maximal discontinuous incremental exercise tests; physical activity using accelerometry combined with a global positioning system; and energy expenditure using the doubly labelled water method. The 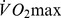 of the male and female adolescents were 73.9±5.7 ml^.^ kg^−1.^ min^−1^ and 61.5±6.3 ml^.^ kg^−1.^ min^−1^, respectively. Total time spent in sedentary, light, moderate and vigorous physical activities per day was 406±63 min (50% of total monitored time), 244±56 min (30%), 75±18 min (9%) and 82±30 min (10%). Average total daily distance travelled to and from school was 7.5±3.0 km (0.8–13.4 km). Mean daily energy expenditure, activity-induced energy expenditure and physical activity level was 12.2±3.4 MJ^.^ day^−1^, 5.4±3.0 MJ^.^ day^−1^ and 2.2±0.6. 70.6% of the variation in 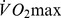 was explained by sex (partial R^2^ = 54.7%) and body mass index (partial R^2^ = 15.9%). Energy expenditure and physical activity variables did not predict variation in 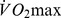 once sex had been accounted for. The highly active and energy-demanding lifestyle of rural Kenyan adolescents may account for their exceptional aerobic fitness and collectively prime them for later training and athletic success.

## Introduction

The success of east African athletes in middle- and long-distance running over the past 40 years raises many questions about why and how this region has produced so many successful runners in endurance events ranging from 800 m to the marathon. As is also the case in Ethiopia [1], the majority of Kenya’s national and international athletes originate from the same geographical district of the country - in the case of Kenya the altitudinous Rift Valley Province - and belong to the same ethnic group - the Nilotic Kalenjin [2]. Among the Kalenjin, the Nandi sub-group have a particularly rich heritage of producing national and international athletes despite accounting for less than 3% (just under one million) of the Kenyan population [2], [3], [4]. Proposed explanations for the phenomenal success of Kenyan runners include genetic, environmental, cultural, socioeconomic, lifestyle and training factors (for reviews see [3], [4], [5], [6], [7]). It is widely accepted that some varied combination of these factors contributes to the success of these athletes, but efforts to investigate, let alone partition the variance, is challenging because sporting success is influenced by both polygenic and environmental factors - some physical and some socioeconomic and cultural.

In terms of physiological factors, maximal oxygen uptake (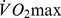), running economy and fractional utilisation of 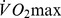 are considered critical factors for endurance running performance [3], [8]. Comparisons of Kenyan and European runners have found that both groups have very high but similar 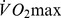 values, but that African runners tend to have a lower oxygen cost of running and therefore a superior running economy [9]. A key question is whether the aforementioned differences between east African and European athletes result from training, or from “naturally” superior running economy in east Africans [10]. Previous studies in untrained (no formal or taught athletic training) Kenyan adolescents have yielded disparate findings. In one study, sedentary male adolescents from Kenya had mean 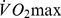 values (47.1±1.3 ml^.^ kg^−1.^ min^−1^; [9]) comparable to their European counterparts (51.6±5.1 ml^.^ kg^−1.^ min^−1^; [11]); whereas, the more physically active Kenyan adolescents had significantly higher values than the sedentary group (62.0±2.4 ml^.^ kg^−1.^ min^−1^; [9]). Similar studies by Larsen et al. [12] also found associations between higher levels of physical activity and superior 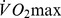 in male Kenyan adolescents, but with 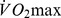 values that were not significantly different from comparable Caucasian populations (mean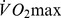 of less active individuals 50.2 ml^.^ kg^−1.^ min^−1^ vs. 55.1 ml^.^ kg^−1.^ min^−1^ of more active individuals). As has been observed with Kenyan athletes, the running economy of untrained Kenyan boys (range 179–246 ml^.^ kg^−1.^ km^−1^) tends to be superior to that reported for untrained Caucasian boys of the same age [12]. The aerobic capacities of untrained Kenyan girls have not been previously reported.

Therefore, this study examined a cohort of male and female adolescents from south Nandi – a rural district of the Kenyan Rift Valley Province responsible for producing many elite Kenyan endurance runners. The study aims were to objectively measure cardiopulmonary fitness, as well as to quantify physical activity levels, distance travelled to school and daily energy expenditure in these pupils to examine any relationship that might exist, as previously found, between 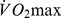 and measures of physical activity and energy expenditure. It was hypothesised that increased physical activity levels and daily energy expenditure (DEE) would be positively associated with individual variation in 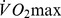.

## Materials and Methods

### Ethics Statement

Both children and parent(s) gave verbal informed consent to participate in the study, which was approved by the Institutional Research Ethics Committee, Moi University, Eldoret, Kenya. This was achieved and witnessed by the principle investigators (Dr. Alexander Gibson, Mr. Robert Ojiambo – who spoke the local dialect – and Dr. Yannis Pitsiladis) with the help of the children’s teachers to provide information and answer questions. Written informed consent was not collected from parents due to illiteracy. Agreement by verbal consent was documented next to each child’s name. This process was in line with the approval received from the local ethics committee.

### Participants

Thirty Kenyan children known by the games teacher to have good cardiopulmonary fitness levels were selected to take part in the study. None of the subjects took part in formal or taught athletic training but all were habitually active. All commuted to and from school five days a week, four times a day (once in the morning, home and back for lunch, then home again in the evening, as was customary) over very undulating terrain. This was completed on foot, as always, with a mixture of walking and running. The number of participants was based on previous related studies as well as budget constraints given the use of the relatively expensive doubly labelled water method to objectively assess DEE and as such, subject numbers are in line with previous studies [12], [13]. Post-hoc sample size calculation revealed that a minimum of 37 subjects was required to obtain a statistical power of 0.8 with an effect size of 0.33. The cohort was made up of an equal number of boys and girls between the ages of 10 and 17. Subjects were recruited from a Kalenjin primary school (Pemja Union School) situated at an altitude of approximately 2400 m on the Nandi Escarpment, south Nandi district, Kenya. The school was identified by a local Kalenjin guide due the school’s rural location in South Nandi and close proximity to a running track for exercise testing. Prior knowledge of the aerobic fitness and physical activity levels of the study population was unknown. The local guide was also blind to the specific aims and objectives of the study when identifying the school and study population. Key physical characteristics of the subjects are summarized in Table 1. In the present study focus on the indicators of cardiopulmonary fitness including 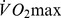 in this highly active cohort is made and findings related to the Kenyan running phenomenon. This data was generated as part of a much larger project involving a unique group of adolescents. The relationships between physical activity, energy demands and adiposity indices of this cohort in relation to obesity and the concept of an energy expenditure ceiling are described in detail elsewhere [14]. Selected pupils at this school (including some of the pupils investigated in the present study) participated in a study investigating the foot strike patterns and collision forces of these habitually barefoot pupils [15].

**Table 1:** Summary of subject ages and anthropometric measures (height and mass).

Sex	n	Age (years)	Mass (kg)	Height (cm)	BMI (kg^.^ m^−2^)
Male	15	13.9±1.6	39.2±9.2	157.6±12.8	15.5±1.4
		10–16	24.5–53.0	132.0–173.0	13.6–18.0
Female	15	13.9±1.2	42.2±7.7	157.0±8.6	17.0±2.2
		11–17	28.0–57.0	134.5–172.5	14.6–22.0

Mean values ± SD and ranges are presented.

### Experimental Design

The age of each subject was reported in years. Height was measured to the nearest 1 cm and mass to the nearest 100 g, whilst barefoot and wearing the school uniform (a light shirt and either shorts or a skirt). Each child completed two maximal exercise tests as well as a week of simultaneous assessment of physical activity levels and DEE using accelerometry and doubly labelled water. Distance travelled to school (by running and walking) was objectively measured using a global positioning system (GPS) device (Timex Trainer V, Timex Group, Connecticut, USA). Each exercise test was separated by at least 7 days and did not occur during the period of assessment of activity levels and DEE. All testing took place at an altitude of approximately 2400 m. The mean ambient temperature during testing was 22.5±1.8°C and mean relative humidity 55.9±8.8%.

### Exercise Testing

Subjects performed two identical maximal running tests on an outdoor running track. The track was constructed to traditional 400 m track dimensions with the accepted constraints and imperfections of doing so in rural Kenya. As such the track, surfaced primarily with irregular tree bark chip, contained significant undulations (≤5% gradient) especially around the bends. A discontinuous incremental protocol involving three-minute bouts of exercise followed by three-minute bouts of active recovery was used. Such protocols are generally well tolerated by children and typically produce reliable and valid 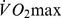
data [16], [17]. Running speed was controlled by having subjects run on the outside of a cyclist on a pushbike using a calibrated speedometer. This method was considered safer for the adolescents involved but meant that they would have to run faster on each bend to keep pace with the bike on the inside. Variance in pace was also an accepted limitation under the constraints of the track and pacing strategy. For accurate measurement of running velocity, each subject also wore a GPS device (GPSports Systems, Fyshwick, Australia). Subsequent to a short warm up period and habituation to the equipment, subjects began with a three-minute walk at 6 km^.^ hr^−1^. Subjects then went immediately into a slow jog at 8 km^.^ hr^−1^ for three minutes before returning back to a three-minute walk at 6 km^.^ hr^−1^. After each succeeding three-minute walking-rest bout, subjects then continued with a three-minute run that was 2 km^.^ hr^−1^ faster than the previous attained speed until volitional exhaustion. The running test was terminated when the subject stopped or when the subject began to consistently fall behind the rear wheel of the bike despite verbal encouragement. Tests were considered acceptable if maximum heart rate was either ≥95% of the predicted value (220 minus age in years) or if the subject showed signs of intense effort including breathlessness, sweating, facial flushing, unsteady gait or difficulty maintaining pace as is customary in paediatric exercise testing [16].

Cardiopulmonary variables were measured continuously throughout each test. Breath-by-breath gas analysis was conducted using a validated portable indirect-calorimetry system connected to a facemask (Cosmed K4b^2^, Cosmed, Italy). The gas analysis system was calibrated before and after each exercise test using ambient air and a gas of a known concentration (O_2_ 16.0%, CO_2_ 6.0%, BOC Kenya Ltd., Nairobi, Kenya), the turbine flow meter using a three litre syringe, and time-delay by an experimenter breathing synchronously into the device. The device was worn over the shoulders of each individual so that the battery and gas analyser lay on the anterior and posterior aspects of the child’s thorax, respectively. This device has been shown to yield accurate and repeatable measurements of rate of oxygen uptake (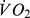) and carbon dioxide elimination (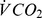) [18], [19] and has previously been used in a similar population with comparable methods and conditions [13]. Heart rate was recorded by the same device using a telemetric Polar heart rate monitor chest strap (Polar Electro Oy, Kempele, Finland). The combined weight of the equipment carried by each child during testing was approximately 1200 g. The addition of up to 10% of total body mass to the trunk has been shown to be well tolerated without significantly affecting 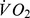 [20] and therefore this additional weight was not incorporated in the study calculations.

### Physical Activity Levels and Distance Travelled to School

Physical activity levels and patterns were objectively measured for 7 consecutive days during school term time using one of 10 ActiTrainer uni-axial accelerometer (Firmware version 7.5.0, ActiGraph LLC, Pensacola, FL, USA) with similar previous levels of use. The device was attached to the right hip of each subject using an elasticized belt and adjusted to ensure a tight fit. Each child was instructed to wear the device at all times except when sleeping and when bathing. The recording epoch was set at 15 s and data reduction and analysis is described below. The monitoring of activity levels was considered acceptable if a minimum of 12 h of recordings took place per day for at least six days (to include at least one Saturday or Sunday). Distance travelled to school was measured on a single occasion by asking each child to wear the GPS device along their usual route to and from school during their lunch break. Data was then multiplied by two in order to reach the total distance travelled to and from school each day.

### Daily Energy Expenditure

Doubly labelled water was used for the objective assessment of DEE over the same seven days used to quantify physical activity levels. Doubly labelled water was supplied by the Energetics Research Group, part of the Institute of Biological and Environmental Sciences at the University of Aberdeen, (Aberdeen, UK) where subsequent analysis was also completed. The protocol involved the oral consumption of doubly labelled water with 0.15 g H_2_O^18^ and 0.12 g ^2^H_2_O per kg body mass [21]. Urine samples were collected from each subject prior to dosing and every day thereafter, morning and evening, for 7 consecutive days. Samples were then frozen in duplicate, along with local water samples, prior to analysis by mass spectrometry. Equation A6 of Schoeller et al. [22] was used to evaluate dilution space and to estimate the rate of carbon dioxide production from the differential disappearance of the 2 isotopes based on multi-point elimination curves. This was converted to an estimate of energy expenditure using the Weir equation [23]. The Schofield equations [24], based on age and gender, height and body mass, were used to predict basal metabolic rate (BMR) for this calculation. DEE, activity-associated energy expenditure (AEE; 0.9 X TEE – BMR) and physical activity level (PAL; TEE/BMR) are reported.

### Analysis and Statistics

Accurate running speeds were calculated by averaging GPS data across each three minute running bout for each child (data not included). Cardiopulmonary variables from the last 30 s of each exercise bout were edited by removing data values that fell outside of three standard deviations from the mean (indicating ‘false’ breaths often caused by an interruption to the breathing cycle such as coughing or swallowing) and averaged and plotted on the same axes. The 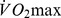 reported was determined from both tests by taking the highest congruent value that lay on linear plots of both incremental tests. The anaerobic threshold was determined by the V-slope method as described by Beaver et al. [25]. Running economy was calculated using the last 30 s of the 10 km h^−1^ loaded exercise bout to allow comparison with Kenyan pupils measured by Larsen et al. [13]. For both anaerobic threshold and running economy the mean of the two test values was taken. Means, standard deviations and ranges are reported for each sex.

Accelerometer data were analysed using algorithms developed in R (version R 2.9.0., R Foundation for Statistical Computing, Vienna, Austria; http://www.R-project.org). Briefly, R was programmed to re-integrate data collected in 15 s epochs to 60 s; edit the data to exclude periods where it was likely that the accelerometer was not being worn [26] and provide summary statistics. The output generated by R included: counts per day (CPD), total monitoring time, number of minutes spent in sedentary activity and in each physical activity intensity (i.e. light, moderate, vigorous and moderate to vigorous physical activity (MVPA)), as defined by the recommended [27] Evenson’s cut-points [28] and percentage of overall time spent in the specified activity level. The use of the Evenson cut-points are in line with recent recommendations by Trost et al. [27] who argue that these cut-points provide acceptable classification accuracy for the different physical activity intensities in children between 5 and 15 years of age.

Data is expressed as mean ± SD and range following a Shapiro-Wilk test of normality**.** Time spent in sedentary and vigorous activities was not normally distributed, however, time in light, moderate and MVPA was. The relative influence of sex, height, activity levels and patterns, distance travelled to school and DEE on 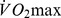 were assessed using a model comparison approach involving regression analysis on SPSS software (SPSS, Version 17.0, SPSS, Chicago, IL, USA). Significance was declared at p<0.05.

## Results

### Subject Ages and Anthropometric Measures

The ages and physical characteristics of this population are presented in Table 1. The mean age for both males and females was 13.9 years. Females were slightly more homogenous in terms of height and mass but considerable variation still existed. The girls were heavier than the boys (42.2±7.7 vs. 39.2±9.2 kg) yet the two sexes were similar in height so that the girls had a higher body mass index (BMI) than the boys (17.0±2.2 vs. 15.5±1.4 kg^.^ m^−2^) as would be expected in this circumpubertal age range.

### Peak VO_2_, Anaerobic Threshold and Running Economy

Mean 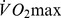, anaerobic threshold and running economy for each sex is presented in Table 2. All subjects achieved 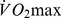 as determined using the criteria previously stated. The first maximal exercise test completed by one of the boys failed due to technical problems and hence single values were used for his assessment. Mean running velocity recorded by GPS (expected) were: 5.0±0.5 km^.^ hr^−1^ (6 km^.^ hr^−1^), 7.6±0.6 km^.^ hr^−1^ (8 km^.^ hr^−1^), 9.4±0.7 km^.^ hr^−1^ (10 km^.^ hr^−1^), 11.0±0.6 km^.^ hr^−1^ (12 km^.^ hr^−1^), 12.5±0.7 km^.^ hr^−1^ (14 km^.^ hr^−1^), 14.4±0.6 km^.^ hr^−1^ (16 km^.^ hr^−1^), 16.2±0.8 km^.^ hr^−1^ (18 km^.^ hr^−1^) and 18.2±0.1 km^.^ hr^−1^ (20 km^.^ hr^−1^). Final running velocity ultimately achieved was between 14.3–18.3 km^.^ hr^−1^. The boys reached higher maximal running velocities than the girls on average (16.5±1.4 vs. 15.8±0.9 km^.^ hr^−1^). The 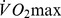 of the boys averaged 73.9±5.7 ml^.^ kg^−1.^ min^−1^ and the girls 61.5±6.3 ml^.^ kg^−1.^ min^−1^. 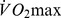 at the anaerobic threshold for both sexes was 2.0 l^.^ min^−1^ (2.02±0.49 for the boys and 1.96±0.32 for the girls). This corresponded to an average of 51.5±3.7 ml^.^ kg^−1.^ min^−1^ or 70.0±6.5% of 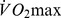 in the boys and 46.9±6.6 ml^.^ kg^−1.^ min^−1^ or 75.2±5.8% of 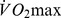 in the girls. The average running velocity of the children when paced at 10 km^.^ hr^−1^ was 9.4 km^.^ hr^−1^ when analysed using GPS. The mean running economy of the girls and boys at this pace was 256.7±29.3 ml^.^ kg^−1.^ km^−1^ and 270.7±21.0 ml^.^ kg^−1.^ km^−1^, respectively.

**Table 2:** 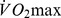
, anaerobic threshold and running economy in the 30 untrained Kenyan school children.

	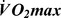 (l^.^ min^−1^)	(ml^.^ kg^−1.^ min^−1^)	(ml^.^ kg^−0.75.^ min^−1^)
Boys	2.88±0.64	73.9±5.7	183.8±15.0
	1.97–4.02	63.9–81.6	157.3–213.6
Girls	2.61±0.31	61.5±6.3	156.4±10.7
	1.93–3.06	47.9–68.9	131.6–170.3
	**Anaerobic Threshold**		
	**(l^.^ min** ^−**1**^ **)**	**(ml^.^ kg** ^−**1.**^ ** min** ^−**1**^ **)**	**(% max)**
Boys	2.02±0.49	51.5±3.7	70.0±6.5
	1.40–2.80	46.6–57.1	58.7–79.8
Girls	1.96±0.32	46.9±6.6	75.2±5.8
	1.25–2.35	36.0–58.7	64.8–85.2
	**Running Economy**		
	**(ml^.^ kg** ^−**1.**^ ** min** ^−**1**^ **)**	**(ml^.^ kg** ^−**1.**^ ** km** ^−**1**^ **)**	
Boys	45.1±3.5	270.7±21.0	
	37.9–52.0	227.5–312.0	
Girls	42.8±4.9	256.7±29.3	
	36.6–54.3	219.9–325.6	

Absolute values are given for 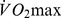 along with 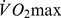 expressed in the traditional ratio to body mass and body mass^0.75^ using allometric scaling. Mean values ± SD and ranges are given.

### Activity Levels and Distance Travelled to School

Accelerometer data for the group is presented in Table 3. All participants met the requirements for acceptable monitoring as documented above. The 30 children accumulated an average of 1148±244 counts per minute (CPM) or 932±204×10^3^ CPD over an average recording period of 14±4 hours per day. The daily mean time spent in sedentary activity was 406±63 minutes or 50% of total recording time. Total time spent in light physical activities was 244±56 min (30% of total monitored time); moderate activity, 75±18 min (9%); vigorous, 82±30 min (10%) and therefore MVPA, 156±33 min (19%). The cumulative daily distance travelled to school by each sex is presented in Table 3. Substantial variation was seen in the total distance required to travel between home and school each day in both sexes. The mean total daily distance travelled to and from school for the group as a whole was 7.5±3.0 km (0.8–13.4 km). Males travelled a total 8.9±2.8 km on average each day compared to the girls who travelled 6.2±2.6 km. Seven (47%) of the boys travelled over 10 km each day whereas only two (13%) of the girls did the same. Four (27%) of the girls travelled less than 5 km each day whereas only one (7%) of the boys lived so close.

**Table 3:** Daily physical activity, total daily distance travelled to school and daily energy expenditure characteristics in the 30 untrained Kenyan school children.

	Mean ± SD	[range]
kCPD	932±204	[664–1484]
CPM	1148±244	[809–1843]
Sedentary (min)	406±63	[328–561]
Light (min)	244±56	[132–354]
Moderate (min)	75±18	[43–113]
Vigorous (min)	82±30	[32–153]
MVPA (min)	156±33	[109–234]
% time sedentary	50	[39–67]
% time light	30	[17]–[42]
% time moderate	9	[5]–[17]
% time vigorous	10	[4]–[19]
% time MVPA	19	[14]–[30]
Daily distance to school (km)	7.5±3.0	[0.8–13.4]
TEE (MJ^.^ d^−1^)	12.2±3.4	[7–20.7]
PAL	2.2±0.6	[1.3–3.5]
REE (MJ^.^ d^−1^)	5.6±0.6	[4.4–6.8]
AEE (MJ^.^ d^−1^)	5.4±3.0	[1.2–12.7]

kCPD - kilo counts per day.

CPM - Counts per day.

MVPA - Moderate to vigorous physical activity.

TEE - Total energy expenditure.

PAL - Physical activity level.

REE - Resting energy expenditure.

AEE - Activity-associated energy expenditure.

Mean values ± SD and ranges are given.

### Daily Energy Expenditure and Regression Analysis of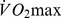, Energy Expenditure Data and Physical Activity Data

DEE recorded in this cohort was also at the highest end of the physical activity continuum with mean DEE, AEE and PAL for the Kenyan adolescents of 12.2±3.4 MJ^.^ day^−1^, 5.4±3.0 MJ^.^ day^−1^ and 2.2±0.6 respectively (Table 3). In regression analysis of the data using sex, age, height and mass 54.7% of the variation in 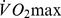 was explained by sex (p<0.001). Height, mass and BMI significantly predicted 8.1%, 15.0% and 15.9% of the variation respectively. Height and mass were highly correlated (R^2^ = 0.86) and therefore BMI was used as our best-fit model. Hence 70.6% of the variance in 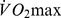 was shared with sex and BMI. The standard error of the estimate (SEE) of 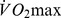 in this model was 4.8 ml kg^−1^ min^−1^ or 7.1% of the mean 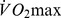 (Table 4.). Energy expenditure and physical activity variables, including daily distance travelled to school, did not contribute to improvements in this model.

**Table 4:** 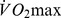
 prediction models.

Dependent	Independent variable	Coefficient variable	P	Partial R^2^	R^2^	SE(ml^.^ kg^−1.^ min^−1^)
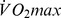	Intercept	86.5				
	Sex	9.06	<0.001	54.7%		
	BMI	−1.98	<0.001	15.9%	70.6%	4.83

### Comparison between Boys and Girls

Boys achieved significantly higher 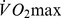 values, were significantly more active and travelled greater distances to school than the girls, however, no significant difference in energy expenditure variables were observed between genders (data not included) and there were no correlations between inter-individual variation in 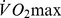 and energy expenditure and physical activity variables after accounting for gender.

## Discussion

In this study, 15 Kenyan boys and 15 Kenyan girls completed two maximal exercise tests on an undulating outdoor running track. On both occasions, aerobic variables including 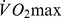, ventilatory anaerobic threshold and running economy were determined. The activity levels, daily distance travelled to school and DEE of each child were also objectively measured. Both male and female students reached high 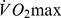 values of 73.9 and 61.5 ml^.^ kg^−1.^ min^−1^, respectively. Comparing 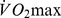 values between adolescent populations is problematic as it dependent on subject selection procedures and exercise protocols and significantly influenced by growth, maturation and development (for reviews see [29], [30]). Nevertheless, 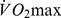 values in the present study were significantly higher than the values previously reported in Kenyan and Caucasian populations. The first study that compared the aerobic capacity of Kenyan and Caucasian runners reported the 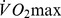 of untrained active (16.8 years, n = 4) and sedentary (14.2 years, n = 6) urban Kenyan boys [9]. The active boys demonstrated a higher 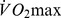 than the sedentary boys; 62.0 vs. 47.1 ml^.^ kg^−1.^ min^−1^; a difference of 32% [9]. In line with these results are findings from a study by Larsen et al. [12] that examined the anthropometrics, 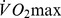, running economy and physical activity levels of Nandi town (16.6 years, n = 11) and village boys (16.6 years, n = 19). The village boys had a mean 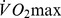 of 55.1 ml^.^ kg^−1.^ min^−1^ and the town boys averaged 50.2 ml^.^ kg^−1.^ min^−1^ [12]. Similar findings and conclusions were published by Larsen et al., in 2005 [13] when comparing Nandi town and village boys. The 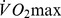 values of the sedentary boys reported by these authors were similar to those of European populations (50.3 ml^.^ kg^−1.^ min^−1^; [13]), whereas the active village boys demonstrated a 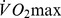 that was approximately 10% higher (56.0 ml^.^ kg^−1.^ min^−1^; [13]) [11], [31], [32], [33]. Average 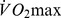 reported in European populations for ages between 10.2 and 16.3 years are between 47.2 and 60.0 ml^.^ kg^−1.^ min^−1^ for boys and 36.9 and 53.6 ml^.^ kg^−1.^ min^−1^ for girls [31], [33], [34], [35]. Similar ranges have been reported in American and Chinese cohorts [36], [37]. Even trained, high-achieving distance runners of similar ages in the USA [38] did not achieve the high values attained in the present sample of Kenyan adolescents. The 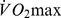 values of the present study are even more remarkable if one considers that these adolescents were tested at an altitude of approximately 2400 m. The differences in 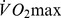 observed in our study and those previously reported in male, Kenyan adolescents [12], [13], [9] may be due, in part at least, to the fact that the earlier studies used motorised treadmills to elicit maximal aerobic capacity. Despite efforts to achieve familiarity with treadmill running, treadmill running is particularly foreign in these rural populations and can therefore attenuate maximal values. Our high 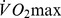 values may also partially reflect the very low body mass of the subjects tested in this study (Table 1). The implications of low body mass and scaling 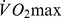 to body mass to achieve a size-free variable is subject to considerable debate in the literature [39], [40] and therefore outside the scope of the present paper. 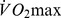 values have thus been presented as absolute values; as a ratio standard, per kilogram of body mass (ml^.^ kg^−1.^ min^−1^) and using allometric scaling, bodymasŝ^0.75^ (ml^.^ kg^0.75.^ min^−1^) to allow comparison with the relevant literature. Whilst no relationship between activity levels, energy expenditure and 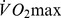 has been demonstrated in this population, as will be more fully discussed, this cohort was very active as is demonstrated in their high CPM and PAL values as well as some of the distances commuted to and from school. Whilst no formal or taught exercise took place, to label the group untrained would be misleading. Although the previous studies in Kenya [9], [12], [13] did not measure activity levels and daily energy expenditure, as this study has, some of the differences in results may be as a result of a more active population measured here whose ‘training’, with no specific goal, was in the context of a challenging daily commute to and from school.

The anaerobic threshold of the subjects in this study occurred at a high percentage of their 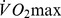. On average the anaerobic threshold of the boys occurred at 51.5±3.7 ml^.^ kg^−1.^ min^−1^ or 70.0±6.5% of their 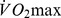. In the girls the same threshold occurred at 46.9±6.6 ml^.^ kg^−1.^ min^−1^ or 75.2±5.8% of their 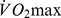. Compared to European adolescents, the anaerobic thresholds reported here are also high. In untrained Belgian adolescents between the ages of 10 and 17 years, anaerobic thresholds ranged between 57.9 and 67.6% of their 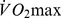 for males and 53.8 to 67.8% for females [41]. These high percentages of 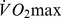 may reflect the higher physical activity and DEE levels of our subjects. One study in an adult population found that this threshold occurs at 61.6% of their 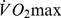 in untrained individuals and 68.8% 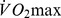 in trained [42].

The mean running economy recorded for boys and girls in the current study was 270.7±21.0 and 256.7±29.3 ml^.^ kg^−1.^ km^−1^, respectively. The boys in Larsen et al. [12] paper had a mean running economy of 221.1±17.9 ml^.^ kg^−1.^ km^−1^ and were therefore more efficient runners than this sample. This may be accounted for by two study limitations. The first being the undulating nature of the outdoor running track used in the present study and the second, the varying nature of running speed as reflected in the recorded GPS. Such variations in gradient and running velocity will have resulted in failure to maintain exact work rates and may partially account for the lower running economy observed in our tests. Our subjects also had a lower mean age (13.9 vs. 16.6 years) and a wider range of ages (10–16 years vs. 15.2–18.4 years) compared to those studied by Larsen et al. [12]. It is well established that running economy improves with chronological age and our subjects demonstrated running economy comparable to subjects of a similar age previously studied [43], [44].

The differences observed between the Kenyan village adolescents and their urban and European peers in previous studies has often been attributed to greater physical activity level observed in the rural population, especially regular walking and running to and from school [12]. The same conclusion has been drawn when comparing the 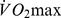 of adolescents in two European countries that differed in physical activity levels [34]. Running long distances daily to and from school as children and adolescents has long been thought to be a contributor to the success of East African runners [5]. In addition to the work cited above, recent studies in both Kenya and Ethiopia supports the association between athletic performance and daily running and walking [1], [2].

The Kenyan boys and girls spent 40% of their recorded time engaged in light and moderate activity and only 10% of their recorded time engaged in vigorous intensity activity. Although this breakdown is largely consistent with previous findings in other populations [45], [46], this sample of adolescents spent more time engaged in vigorous activity than previously reported in representative samples of European and American populations between the ages of 6 and 19 years. In concurrent work carried out using the same population, the relationship between physical activity and energy demands was examined [14]. Body mass, average accelerometer counts per minute (a measure of volume of physical activity) and time in light activities predicted 46% of the variance in DEE (p<0.05; SEE 6.4 MJ^.^ day^−1^) and AEE accounted for 44% of the DEE. Other qualifiers of physical activity intensity and distance travelled to school were not related to variation in DEE, AEE or PAL. Higher PALs, DEE and AEE were found compared to Western populations, which opposes the concept of a ceiling of energy expenditure [14], [47], [48].

The evidence relating habitual physical activity to 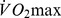 in children and adolescents is equivocal [49]. High levels of physical activity have been correlated only modestly with high 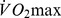 values in only some studies [41], [50]. Such differences might be expected because the physical activity patterns of most children and adolescents are typically sporadic and short in nature and, therefore, may not contribute to improvements in aerobic fitness if indeed causality exists [51]. The effect of lifestyle factors such as physical activity on developmental changes in aerobic power may also be confounded by neglecting to account for changes secondary to the processes of growth, development and maturation that may mask or outweigh external influences. Additionally previous studies have not always used such objective measures of physical activity and aerobic parameters that accelerometry and indirect calorimetry afford.

Trowbridge et al. [52] reported a weak but significant relationship (r^2^ = 0.18; p<0.05) between 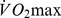 and activity related energy expenditure in young Caucasian Americans but not in African-Americans in the same age group. This difference may have been related to the intensity of exercise in which each population participates. Rutenfranz et al. [34] concluded that active children are 5–10% fitter than untrained children, findings mirrored by work completed in Kenya [9], [12]. Larsen et al. [12] found a positive correlation between physical activity and 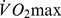 (r = 0.55). In a more recent study, in which these two parameters were measured both quantitatively and accurately, 248 children of both sexes of mean age 9.8±0.6 years 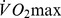 was weakly correlated with mean accelerometer counts (r = 0.23 in both sexes) and time spent in vigorous activity (r = 0.32 and 0.30) measured over four days using accelerometry and Trost cut-points [53]. The authors concluded that approximately 10% of the variability in 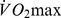 was attributable to differences in both mean daily physical activity levels and time spent in vigorous physical activity [53]. Of course there is an inferred causality in these assumptions and perhaps fitter children are more active as a result of the increased capacity for physical activity.

Cardiopulmonary fitness is a complex phenotype to study. Undoubtedly inter-individual variation in 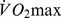 and its trainability is likely to be influenced by both genetic and environmental influences and interaction [52], [54], [55], [56]. There is some evidence that the pre-training level fitness and childhood patterns of physical activity may also have an important role in the modulation of later responses to exercise [57], [58]. No correlations between inter-individual variation in 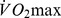 and energy expenditure and physical activity variables were found after accounting for gender. This finding may be due to the small number of subjects with heterogeneous anthropometric characteristics, age and maturity status, and high 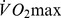, activity levels and energy expenditure.

A limitation of the current study is that growth, maturity status and lean body mass were not assessed. It was evident, subjectively, that the adolescents participating in this study were both pre-pubescent and at varying stages of puberty as one would expect in a mixed sample of girls and boys between the ages of 10 and 17. Thus in this study we can only compare the chronological ages of each child, which were only deduced by self-report. Another caveat when interpreting the data was the impossibility of quantifying and maintaining exact work rates throughout the discontinuous, incremental protocol that we used. This was due to method of pacing and an undulating track surface. Despite this, incremental rises in 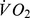 were observed with each test and using GPS to calculate a more accurate average running velocity allowed us to more accurately quantify running economy. However, running economy estimations may have been particularly affected by the undulating track surface and the non-consistent running speed as previously discussed. The authors also acknowledge that the portable indirect-calorimetry system (Cosmed K4b^2^, Cosmed, Italy) used is sometimes unreliable, particularly when used outdoors and requires frequent and careful calibration. Whilst calibration was conducted frequently by accustomed users of the device and whilst the device had been validated by the team, indoor and outdoor, in the U.K. [18], the authors recognise that replication of results is needed to confirm the cardiorespiratory data. Notably, the introduction of an automatic, periodical recalibration during sampling has gone someway in an attempt to improve the reliability and validity of the device. In addition to this the authors acknowledge that the optimal accelerometry cut-point for measurement of MVPA remains to be established, and may vary between populations and between accelerometer models. While the error in estimation of MVPA is therefore unclear in the present study, a widely used accelerometer cut-point [28] was adopted and it is likely that any bias in measurement applied across the sample. Despite these potential sources of error neither effect the conclusions of this study and both must be considered in context of the potential pitfalls in the use of alternative methodology.

The generalisation of such data in this population is of course unknown without further study. Given previous published studies [9], [12], [13], it is unlikely that the study cohort is representative of adolescents in the South Nandi region of Kenya or indeed the wider east African region. Similar aerobic fitness and physical activity levels would be expected in other pupils of the school if tested; a reflection of the schools exceptional rural African setting (i.e. located close to the summit of Nandi Rock, at the end of and overlooking the escarpment). The school was not famed for developing athletes and no pupil took part in formal athletic training. Whilst some selection bias cannot be excluded, the school was identified without prior knowledge of the cardiopulmonary fitness and physical activity levels of the study population and blind to the aims and objectives of the study.

### Conclusions

We describe a population of Kenyan adolescents of both sexes who have demonstrated an enormous capacity for physical activity. Physiological markers of cardiopulmonary fitness, activity levels, distance travelled daily to school and DEE were quantified. 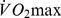 and anaerobic threshold are larger than previously documented in any paediatric population we are aware of. Activity levels and DEE are also exceptionally high. Whilst the relationship between physical activity and cardiopulmonary fitness remains incompletely understood, and whilst maturation and scaling issues complicate the picture, this data has demonstrated no correlation between activity levels, energy expenditure and 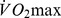 within this group. However, when analysed in the context of other groups of adolescents previously studied the comparatively high 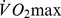 and anaerobic threshold are mirrored by comparatively high measures of physical activity and energy expenditure. The highly active and energy demanding lifestyle of these children, correlated with their high physical fitness, may together prime them for later training and athletic success as well as permit the early identification of talent in this region.
